# Surgeon-Performed Point-of-Care Ultrasound in Complex Breast Lesions: Implications for Oncologic Diagnosis and Surgical Planning

**DOI:** 10.7759/cureus.115072

**Published:** 2026-08-24

**Authors:** Christelle Najib, Hussein Hamze, Ali Hteit, Youssef Finianos, Georges S Nicolas, Rony Bou Rizk, Joel Al Rumhein, Saadeddine Abou Zahr, Aya El Hindi, Manuel Manouchakian, Alexandre El Hajj, Mohamad Tlais, Issam El Fassih, Mohamad I Siblini

**Affiliations:** 1 Radiology, University of Balamand, Beirut, LBN; 2 General Surgery, American University of Beirut Medical Center, Beirut, LBN; 3 General Surgery, University of Balamand, Beirut, LBN; 4 General Surgery, Saint George University Medical Center, Beirut, LBN; 5 Urology, Saint George University Medical Center, Beirut, LBN; 6 General Surgery, Makassed General Hospital, Beirut, LBN

**Keywords:** bi-rads, breast cancer, breast-conserving surgery, breast ultrasound, intraoperative ultrasound, point-of-care ultrasound, surgeon-performed breast ultrasound

## Abstract

Surgeon-performed point-of-care breast ultrasound (POCUS) has become a practical adjunct in the evaluation and management of breast lesions, allowing real-time imaging to be incorporated directly into clinical and operative decision-making. In the oncologic setting, complex breast lesions often require timely characterization, tissue diagnosis, localization, and surgical planning, and the separation of imaging and surgical workflows may contribute to delay, fragmented care, and reduced procedural efficiency. This narrative review examines the technical principles, diagnostic applications, surgical planning utility, clinical outcomes, and implementation requirements of surgeon-performed POCUS in complex breast disease. PubMed, Scopus, and Google Scholar were searched without language restriction, and the retrieved evidence was synthesized thematically. No protocol was registered, and no formal risk-of-bias assessment or quantitative pooling was undertaken. Surgeon-performed POCUS supports immediate assessment of palpable, screening-detected, cystic, solid, and otherwise complex breast lesions using standardized sonographic descriptors and Breast Imaging Reporting and Data System (BI-RADS) interpretation. Limited prospective data indicate diagnostic validity approaching that of radiologist-performed ultrasound when the examination is undertaken by appropriately trained surgeons, although direct head-to-head comparisons remain scarce. In breast-conserving surgery (BCS), predominantly single-institution observational studies have associated intraoperative ultrasound (IOUS) guidance with improved lesion localization, lower positive margin and re-excision rates, and favorable cosmetic outcomes. The technique also facilitates same-visit interventions, including aspiration, core needle biopsy, clip placement, and preoperative localization. Successful adoption requires competency-based training, quality assurance, and institutional credentialing within multidisciplinary breast care models. By linking imaging, biopsy, localization, and operative planning within a single workflow, surgeon-performed POCUS may improve diagnostic efficiency and patient-centered outcomes, although the supporting evidence is largely observational and prospective comparative trials are lacking.

## Introduction and background

Breast cancer remains one of the most prevalent malignancies affecting women worldwide, and the interval between presentation and diagnosis can materially influence the treatment trajectory [1,2]. The conventional diagnostic workflow has been sequential: radiologists performed and interpreted breast ultrasound within dedicated imaging suites, and surgeons awaited formal reports before making operative decisions [3].

Surgeon-performed point-of-care breast ultrasound, referred to variably as point-of-care breast ultrasound (POCUS), surgeon-performed breast ultrasound, or point-of-care breast sonography, has progressively altered this model. Three distinct practices should be separated at the outset. Radiologist-performed diagnostic breast ultrasound is a formal, reported examination conducted in an imaging suite. Surgeon-performed diagnostic POCUS is a focused, question-driven examination conducted by the treating surgeon at the clinical encounter. Intraoperative ultrasound (IOUS) is the use of real-time imaging within the operative field to localize a lesion and assess resection margins [4]. These differ in operator, setting, documentation standard, and, importantly, in the strength of the evidence supporting them. The operating surgeon performs the examination and integrates the sonographic findings with the physical assessment during the same encounter, removing the referral interval and the delay associated with asynchronous interpretation [5,6]. Compact, handheld ultrasound systems have made this approach logistically feasible across a wide range of clinical environments, from outpatient suites to intraoperative settings and emergency departments [7,8]. Same-visit core needle biopsy (CNB), aspiration, and clip placement are now routine capabilities within this framework [8,9].

**Figure 1:**
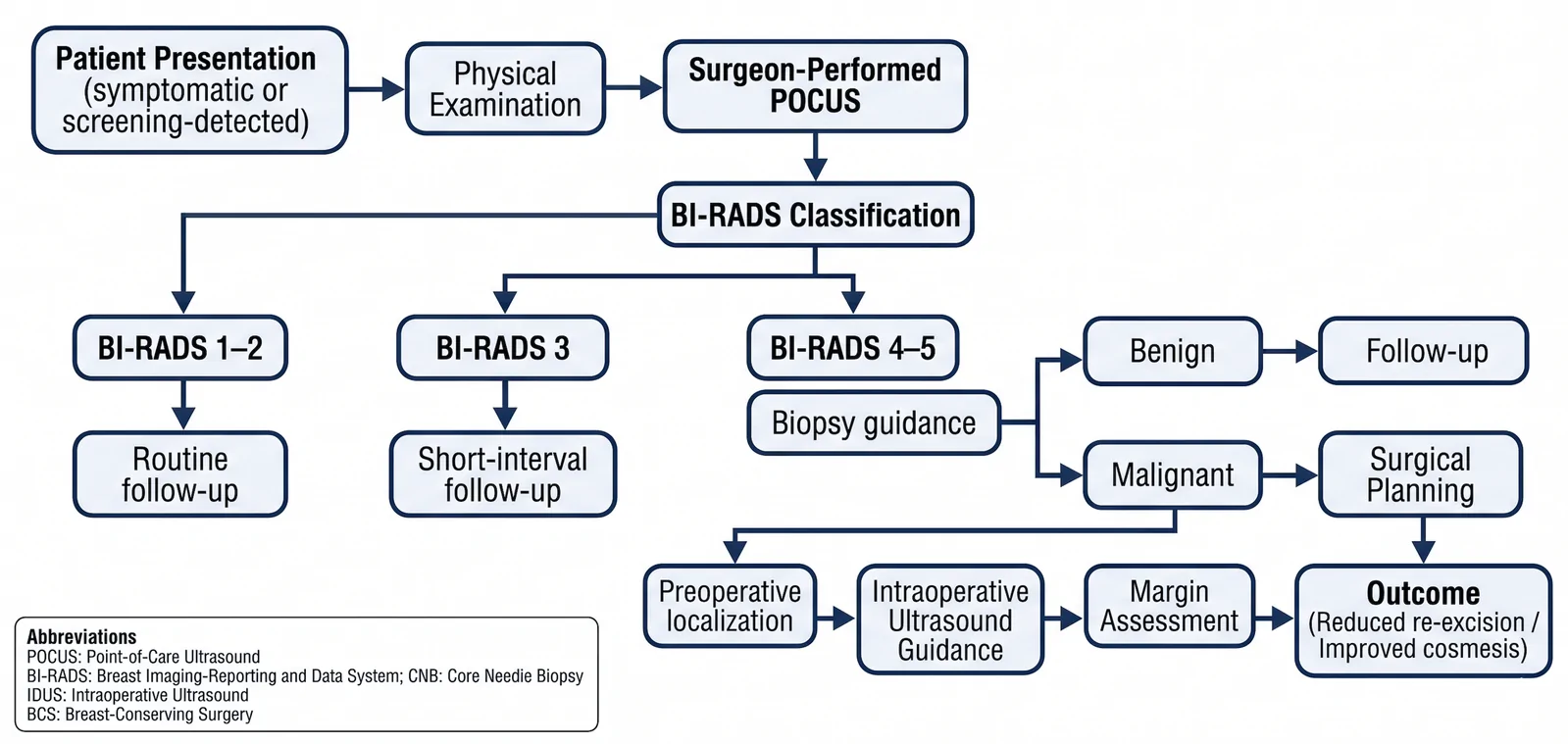
Integrated clinical pathway for surgeon-performed POCUS POCUS: point-of-care breast ultrasound; BI-RADS: Breast Imaging Reporting and Data System; CNB: core needle biopsy; MRI: magnetic resonance imaging; BCS: breast-conserving surgery; IOUS: intraoperative ultrasound Image credit: This figure was created by the authors of this study using Canva. The flowchart illustrates the sequential integration of surgeon-performed POCUS into breast care, from initial patient presentation through diagnostic workup, biopsy guidance, and intraoperative surgical planning

The evidence base across these three practices is asymmetric. Comparative prospective and retrospective series support IOUS guidance during breast-conserving surgery (BCS), whereas evidence for broad surgeon-performed diagnostic POCUS across the full spectrum of complex breast lesions derives largely from single-institution studies with heterogeneous designs. Surgeon-performed POCUS is also operator-dependent, requires structured training and institutional credentialing, and does not replace mammography, magnetic resonance imaging (MRI), or formal radiologic assessment in patients with dense parenchyma, mammographically occult lesions, or suspected multifocal disease. These constraints frame the discussion that follows. The clinical value of surgeon-performed POCUS is particularly evident in the evaluation of complex breast lesions, including palpable masses, spiculated tumors, multifocal disease, inflammatory presentations, and post-traumatic findings, where real-time clinicoradiologic synthesis can directly inform biopsy decisions and surgical planning [10]. The use of the Breast Imaging Reporting and Data System (BI-RADS) [11] ensures that imaging terminology remains standardized and that management pathways remain evidence-based (Figure 1).

Literature search, scope, and review type

This article is a narrative review. PubMed, Scopus, and Google Scholar were searched from 2015 to May 2026 without language restriction. The search combined terms for the operator and setting (surgeon-performed, point-of-care, intraoperative, office-based), the modality (ultrasound, ultrasonography, sonography), and the anatomical target (breast, axilla), together with terms for the outcomes of interest (BI-RADS, CNB, localization, margin, re-excision, BCS, training, credentialing).

The review addresses adults with clinically or radiologically complex breast lesions; the interventions of interest are surgeon-performed diagnostic breast POCUS and IOUS during BCS; the comparators, where reported, are radiologist-performed ultrasound and palpation- or wire-guided surgery; and the outcomes considered are diagnostic accuracy and clinicoradiologic concordance, margin positivity, re-excision, mastectomy conversion, time to tissue diagnosis, cost, and patient-reported outcomes. Population-based screening and staging beyond the axilla are outside the scope of this review.

Because this is a narrative review, a protocol was not registered, eligibility criteria were not prespecified, dual independent screening was not performed, and no formal risk-of-bias assessment or quantitative synthesis was undertaken. Preferred Reporting Items for Systematic Reviews and Meta-analyses (PRISMA) 2020 reporting is therefore not applicable, and no claim of systematic completeness is made [12].

## Review

Technical principles and imaging fundamentals

Ultrasound Physics and Probe Selection

High-frequency linear transducers operating in the 10-15 MHz range are the clinical standard for breast ultrasound and provide the axial resolution required for delineating microinvasive margins, assessing skin involvement, and characterizing small lesions [13]. The trade-off between frequency and penetration depth requires operators to reduce frequency to 7-10 MHz when imaging deep lesions near the pectoral fascia in large-volume breasts [13]. Breast tissue imaging relies on acoustic impedance differences at tissue interfaces: fluid-filled cysts appear anechoic, fibrous tissue and Cooper’s ligaments appear hyperechoic, and most solid breast masses appear hypoechoic relative to surrounding glandular tissue [14,15].

Posterior acoustic features provide additional diagnostic information. Malignant tumors with dense fibrous components characteristically demonstrate posterior acoustic shadowing, whereas simple cysts exhibit posterior acoustic enhancement owing to minimal sound attenuation through fluid [13,15]. Tissue harmonic imaging improves the signal-to-noise ratio by detecting harmonic frequencies generated within tissue, reducing clutter and improving discrimination between complex cysts and solid masses [13].

Standard Scanning Technique

Positioning the patient supine-oblique with the ipsilateral arm elevated compresses the breast tissue, reducing depth and bringing deep structures into clearer sonographic range [14]. A radial scanning approach, with the transducer oriented as a spoke radiating from the nipple, allows systematic interrogation of each ductal segment [16]. Radial scanning is technically advantageous for detecting ductal extension, which directly informs lumpectomy margin planning. The terminal duct lobular unit, the site of origin of most breast carcinomas, appears as a hypoechoic tubular structure enveloped by hyperechoic fibrous stroma on sonography [14]. Familiarity with this anatomy is a prerequisite for reliable interpretation.

BI-RADS Descriptors and Nonmass Abnormalities

Standardized terminology underpins the clinical defensibility of surgeon-performed ultrasound. The American College of Radiology (ACR) BI-RADS ultrasound lexicon provides a shared vocabulary for describing sonographic findings [11]. Malignant masses cluster around irregular shape, nonparallel (taller-than-wide) orientation, indistinct or spiculated margins, marked hypoechogenicity, and posterior acoustic shadowing. Benign masses tend to be oval, parallel, and well-circumscribed, often with posterior enhancement or no posterior features (Table 1) [15].

**Table 1:** Sonographic features of breast masses, benign versus malignant, according to the ACR BI-RADS lexicon ACR: American College of Radiology; BI-RADS: Breast Imaging Reporting and Data System; DCIS: ductal carcinoma in situ This table was created by the authors of this study. The descriptor categories follow the ACR BI-RADS ultrasound lexicon [11], and the benign versus malignant feature assignments were compiled by the authors from the sonographic criteria described by Stavros et al. [15]. The table has not been reproduced or adapted from any previously published table, and no reproduction permission was required

Feature	Malignant	Benign
Shape	Irregular	Oval or round
Orientation	Nonparallel (taller than wide)	Parallel (wider than tall)
Margins	Indistinct, angular, spiculated, or microlobulated	Circumscribed with thin echogenic capsule
Echogenicity	Markedly hypoechoic or heterogeneous	Isoechoic or mildly hypoechoic
Posterior features	Acoustic shadowing	Posterior enhancement or no posterior features
Vascularity on Doppler	Central, disordered, high-velocity flow	Peripheral or absent internal flow
Calcifications	Echogenic foci, which may correlate with DCIS	Absent or coarse

Not all findings present as a discrete mass. Nonmass abnormalities, defined as sonographic findings that disturb normal tissue architecture without forming a defined lesion, require a separate interpretive framework. The Japan Association of Breast and Thyroid Sonology (JABTS) has classified these into five subtypes: ductal abnormalities, which may herald ductal carcinoma in situ (DCIS); hypoechoic areas in the mammary glands; architectural distortion; clustered microcysts; and echogenic foci corresponding to microcalcifications [14,17]. Recognizing these patterns is necessary for any surgeon assessing beyond simple mass evaluation.

Functional Imaging and Emerging Technologies

Power Doppler interrogation of internal tumor hemodynamics identifies high-velocity, disordered central blood flow, a feature of malignant neoangiogenesis, and adds physiologic information to structural imaging [18]. Contrast-enhanced ultrasound, which uses intravenous gas microbubbles as an acoustic contrast agent, characteristically demonstrates rapid centripetal enhancement in malignant lesions and delayed, centrifugal filling in benign lesions [13]. Ultrasound elastography quantifies tissue stiffness; malignant tumors are typically three to six times stiffer than normal parenchyma, and high-grade invasive carcinomas may reach a thirteen-fold stiffness differential [13,18].

Artificial intelligence (AI) is expanding the capability of point-of-care breast imaging. Computer-aided detection, computer-aided diagnosis, and deep learning radiomics pipelines are being validated for lesion localization, benign-malignant classification, and radiogenomic profiling [19,20]. Automated breast ultrasound systems acquire full volumetric datasets using a large transducer paddle, yielding standardized documentation and temporal comparability that handheld scanning alone cannot consistently provide [13].

Documentation and Portable Devices

Handheld wireless devices have decentralized high-resolution breast sonography [9]. Portability does not remove the requirement for documentation discipline. Comprehensive standards require clock-face localization, distance from the nipple, orthogonal three-dimensional measurements, and direct surgical skin marking for intraoperative reference [21]. In low- and middle-income countries (LMICs) where mammography infrastructure is scarce or absent, portable ultrasound frequently serves as the primary diagnostic tool. Pooled data across more than 76,000 examinations demonstrate a sensitivity of 80.1% and a specificity of 88.4% for breast cancer detection using handheld systems [22].

Diagnostic role in complex breast lesions

Palpable Masses and BI-RADS Application

Surgeon-performed POCUS converts the assessment of a palpable mass into an immediate, structured sonographic characterization using BI-RADS descriptors, with diagnostic accuracy that, in studies validating BI-RADS assessment against CNB histopathology, is broadly consistent with that reported for radiologist-performed examination [23,24]. Direct comparative studies of surgeon-performed versus radiologist-performed breast ultrasound are scarce; the strongest available prospective evidence for nonradiologist operators concerns diagnostic validity in palpable masses. The surgeon examining the patient can, within the same encounter, assign a provisional risk category, determine biopsy urgency, and select the appropriate procedural modality [23,24]. Integrating tactile examination findings with real-time sonographic morphology produces a clinical synthesis that an asynchronous imaging report cannot replicate.

Correlation with Mammography and Guidance Selection

Nonpalpable lesions present greater complexity. Screening-detected abnormalities visible on only one modality require deliberate cross-modal correlation, and surgeon-performed POCUS serves as the directed second-look instrument [25,26]. When a sonographic correlate is confirmed, ultrasound-guided CNB becomes immediately accessible and is faster, less costly, and less uncomfortable than the alternatives. When no correlate is identified, stereotactic biopsy is appropriate. Radiologic-pathologic concordance then functions as the quality checkpoint, confirming that the sampled tissue represents the mammographic target [26,27].

Ultrasound-guided semi-automated CNB with specimen radiography can achieve high calcification recovery rates and strong diagnostic performance for suspicious microcalcifications in selected cases [28], which qualifies the assumption that calcification biopsy always requires stereotactic guidance. BI-RADS category 4 lesions span a wide malignancy risk range, from 2% to 95%, and represent 15-34% of all biopsied abnormalities [29]. Histopathologic correlation is therefore a clinical necessity rather than a procedural formality.

Spiculated Masses, Multifocality, and Inflammatory Presentations

Spiculated and irregular masses identified on surgeon-performed POCUS carry high malignant predictive value within the BI-RADS framework and should prompt timely tissue diagnosis followed by radiologic-pathologic concordance review [23,24]. Multifocal disease may exceed what focused point-of-care scanning can reliably map. Breast MRI remains the modality of choice for delineating additional malignant foci, with MRI-guided biopsy or localization reserved for cases in which ultrasound cannot define the full extent of disease [30]. Contrast-enhanced ultrasound has demonstrated diagnostic performance comparable to multiparametric MRI for the characterization of BI-RADS 3-5 lesions in selected head-to-head comparisons [31].

Inflammatory presentations add a further diagnostic layer. Idiopathic granulomatous mastitis (IGM) can mimic malignancy both clinically and sonographically, making tissue diagnosis and structured follow-up indispensable whenever imaging is nonspecific [32]. Post-traumatic sequelae, including fat necrosis, may similarly produce irregular morphology. In these settings, POCUS is most useful for documenting morphologic characteristics and guiding the decision between biopsy and surveillance according to the clinical risk profile. Table 2 summarizes the spectrum of complex lesion types amenable to surgeon-performed POCUS evaluation, along with their characteristic ultrasound features and management implications.

**Table 2:** Complex breast lesions evaluated by surgeon-performed POCUS, with ultrasound features and clinical implications POCUS: point-of-care breast ultrasound; BI-RADS: Breast Imaging Reporting and Data System; CNB: core needle biopsy; MRI: magnetic resonance imaging; US: ultrasound; IGM: idiopathic granulomatous mastitis; DCIS: ductal carcinoma in situ; JABTS: Japan Association of Breast and Thyroid Sonology; FNA: fine-needle aspiration; IOUS: intraoperative ultrasound This table was created by the authors of this study, synthesizing findings from the cited literature [10,14,17,23-26,28,30,32]. It has not been reproduced or adapted from any previously published table, and no reproduction permission was required

Lesion type	Key ultrasound features	POCUS contribution	Management implication
Palpable solid mass	Variable morphology, with BI-RADS descriptors applied	Real-time BI-RADS classification at the point of care	CNB guidance if BI-RADS 4 or above
Spiculated or irregular mass	Irregular margins, acoustic shadowing, nonparallel orientation	High-suspicion pattern recognition and immediate risk stratification	Prompt tissue diagnosis and surgical planning
Multifocal disease	Multiple hypoechoic foci on bilateral assessment	Identification of additional lesion burden and extent mapping	Revision of surgical strategy, with MRI if extent is indeterminate
Nonpalpable mammographic lesion	Variable, and may lack a sonographic correlate	Second-look sonography and correlate identification	US-guided CNB if a correlate is found, stereotactic biopsy if occult
Suspicious microcalcifications	Echogenic foci with a DCIS-pattern ductal abnormality	Identification of a sonographic target for CNB	US-guided CNB if targetable, stereotactic biopsy if not
Inflammatory lesion such as IGM	Irregular hypoechoic areas, hyperemia, possible sinus tracts, nonspecific appearance	Documentation of morphology and identification of malignancy mimicry	Tissue diagnosis required, with clinical follow-up
Post-traumatic change such as fat necrosis	Complex cystic-solid lesion, echogenic foci, irregular margins	Morphologic documentation and risk-stratified assessment	Interval follow-up or biopsy according to risk profile
Axillary lymphadenopathy	Cortical thickening beyond 3 mm, absent fatty hilum, rounded morphology	Nodal morphology assessment and guidance for sampling	FNA or CNB for nodal staging and axillary surgery planning
DCIS in a nonmass pattern	Ductal abnormality of JABTS type 1, with echogenic foci	Extent mapping and identification of the surgical target	MRI for full extent, IOUS for excision guidance

Axillary Assessment and Biopsy Guidance

Axillary ultrasound evaluation holds direct clinical relevance in suspected malignancy, enabling identification of morphologically suspicious nodes, characterized by cortical thickening, absent fatty hilum, rounded configuration, and aberrant vascularity, and guiding preoperative fine-needle aspiration or CNB for nodal staging [10]. Triage of palpable breast findings by minimally trained nonradiologist operators has been evaluated within structured referral systems in resource-limited settings [33]; comparable validation data for nonradiologist axillary assessment are not available. Incorporating axillary assessment into the surgeon-performed POCUS workflow at the initial clinical encounter offers an efficient route to comprehensive preoperative staging in outpatient breast practice.

Role in surgical planning and interventional guidance

Preoperative Sizing, Localization, and Multifocality Assessment

Accurate preoperative tumor sizing and localization are foundational to BCS planning, and surgeon-performed ultrasound provides both in real time [34]. Tumor dimensions, spatial orientation, depth, and proximity to anatomical landmarks are assessed directly at the clinical encounter, informing incision design and excision volume. In palpable infiltrating ductal carcinoma, IOUS has been associated with clear lumpectomy margins [35], and in nonpalpable ultrasound-visible lesions, retrospective comparisons report that ultrasound-guided localization reduces reliance on wire placement while preserving healthy tissue [25]. Bilateral whole-breast scanning increases detection of satellite malignant foci beyond the index lesion and may shift the operative decision toward mastectomy [36].

Retrospective data from BCS outcome series indicate that ultrasound-guided localization offers measurable operative advantages over conventional wire localization for ultrasound-targetable nonpalpable lesions, including improved specimen weight-to-tumor-volume ratios [25]. These findings support systematic POCUS integration into preoperative planning irrespective of whether disease is palpable.

Intraoperative Margin Assessment and Re-excision Reduction

Continuous IOUS guidance during excision enables real-time visualization of the tumor boundary throughout the operative field, allowing immediate targeted re-resection when a margin appears sonographically deficient [3]. Findings are consistent across prospective and retrospective series: IOUS-guided BCS reduces positive margin rates and re-excision procedures compared with palpation-guided surgery [3,34,37].

**Figure 2:**
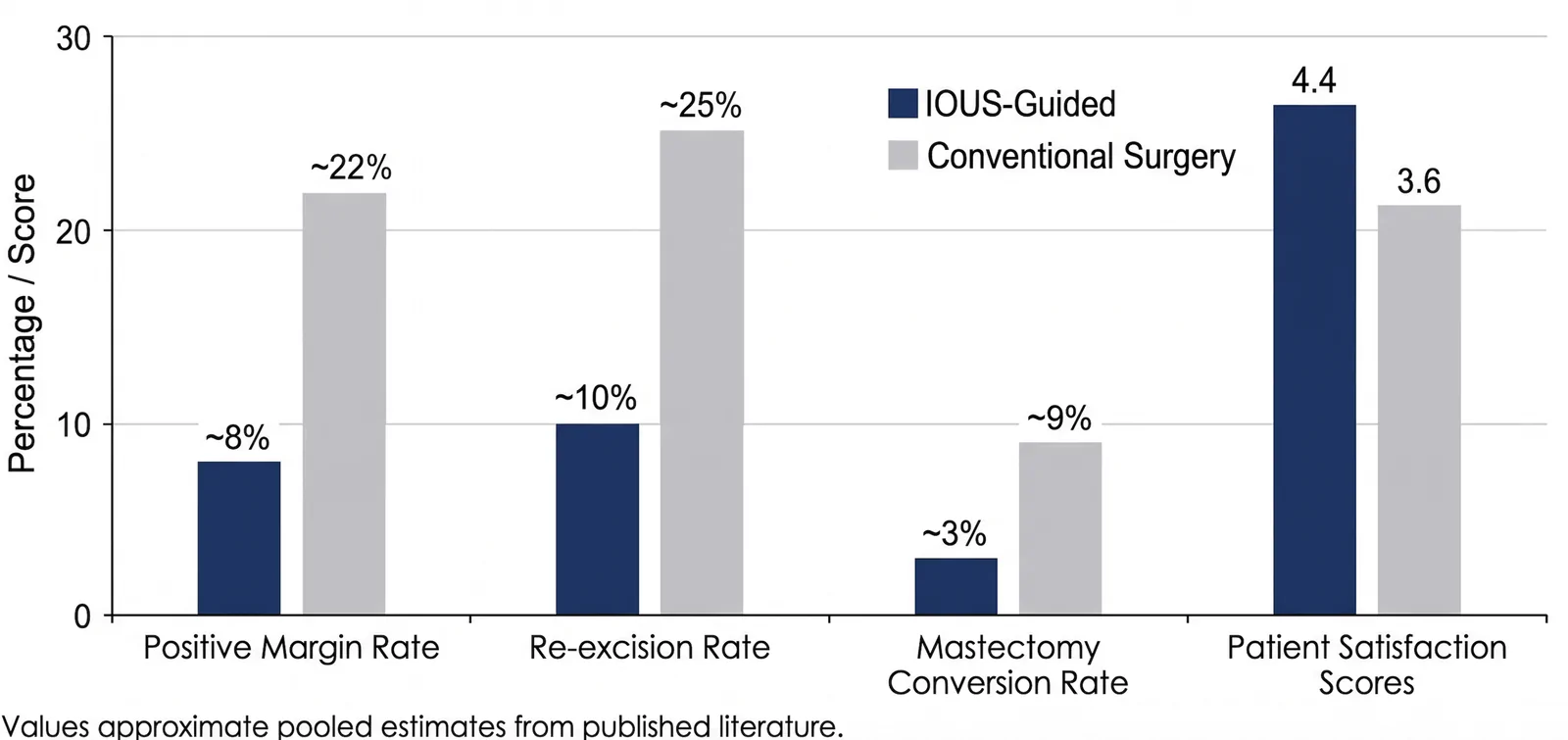
Clinical evidence summary for IOUS-guided breast-conserving surgery outcomes IOUS: intraoperative ultrasound; BCS: breast-conserving surgery Image credit: This figure was created by the authors of this study using Canva. Positive margin rates and re-excision rates are derived from [3,34,37]; mastectomy conversion rates from [3,34]; and patient satisfaction scores from [7,36]. Values are approximate figures drawn from the cited studies. Comparative summary of findings from key studies of IOUS guidance, including positive margin rates, re-excision rates, mastectomy conversion rates, and cosmetic satisfaction scores, for IOUS-guided surgery versus conventional approaches

Karanlik et al. reported lower rates of positive margins and reduced conversion to mastectomy with IOUS-guided BCS in a single-institution series [34]. An independent comparative study of perioperative localization techniques similarly reported improved margin negativity alongside favorable cosmetic outcomes [37]. Both are single-institution studies without randomization, and the observed differences should be interpreted as associations subject to operator selection and learning-curve confounding. Fewer re-excisions translate into fewer repeat general anesthetics, lower procedural morbidity, and better body image [7]. IOUS guidance also reduces operative conversion to mastectomy, preserving breast tissue in patients who would otherwise require more extensive resection (Figure 2) [3].

Axillary Staging

Preoperative axillary ultrasound performed by the operating surgeon identifies sonographically suspicious lymph nodes, defined by cortical thickening beyond 3 mm, absent fatty hilum, or rounded morphology, and guides selective biopsy before definitive surgery [38]. This reduces the likelihood of unexpected nodal findings during the operation and permits more deliberate decisions about the scope of axillary surgery [10,38]. When axillary assessment is incorporated into the surgeon-performed POCUS workflow at the first clinical encounter, preoperative staging can be completed without an additional appointment.

Same-Visit Interventional Procedures

Ultrasound-guided CNB, cyst aspiration, seroma drainage, clip placement for lesion marking, and intraoperative marking needle placement have been validated for diagnostic accuracy and procedural feasibility [39,40]. Prospective data confirm high diagnostic validity for point-of-care breast ultrasound guidance performed by competent nonradiologist clinicians [39]. A single-visit workflow comprising examination, imaging, tissue sampling, and result counseling avoids the fragmented multi-appointment sequences that generate patient anxiety and reduce diagnostic efficiency.

Integration Into Rapid-Access Breast Clinics

One-stop breast clinics represent a logical institutional setting for surgeon-performed POCUS. Large-scale evaluations of these models demonstrate high diagnostic accuracy alongside substantial reductions in time to diagnosis through same-day imaging, biopsy, and clinical assessment [41]. When surgeon-performed ultrasound is embedded within these pathways, imaging and surgical decision-making occur together, handoff delays are removed, care coordination is tightened, and unnecessary admissions decrease.

Clinical outcomes, workflow efficiency, and patient experience

Diagnostic delay carries clinical risk. Surgeon-performed POCUS removes the interval between physical examination and sonographic characterization, compressing a multistep diagnostic sequence into a single clinical encounter [8,9]. In one-stop clinic settings, patients may leave the same day with a sonographic assessment, a guided biopsy, and a provisional diagnosis. This compression reduces the mean time from referral to tissue diagnosis and attenuates the psychological burden of diagnostic uncertainty [41]. For patients presenting with palpable masses, nipple discharge, or concerning skin changes, this immediacy is of particular value.

The efficiency gains extend beyond the patient experience. Fewer clinical visits, higher rates of same-visit tissue diagnosis, and reduced re-excision procedures generate direct cost savings alongside indirect productivity gains [8,21]. 

Transparency is a further consideration. When the surgeon performs the ultrasound during the consultation, the patient can view the screen alongside the clinician and findings are communicated in real time, supporting shared decision-making. Evidence from intraoperative applications shows improved patient satisfaction scores associated with IOUS-guided BCS, attributable to fewer re-excisions, better aesthetic outcomes, and greater operative confidence [7,13]. 

The strength of this evidence base warrants explicit comment. The literature identified consists predominantly of single-institution retrospective series and single-arm or nonrandomized prospective cohorts. No randomized comparison of surgeon-performed with radiologist-performed breast ultrasound was identified, and no randomized trial of IOUS-guided versus conventional BCS was identified with adequate power for margin outcomes. Operator selection, institutional case mix, and learning-curve effects confound comparative estimates, and margin definitions are heterogeneous across studies conducted before and after the Society of Surgical Oncology-American Society for Radiation Oncology consensus on margin adequacy, limiting cross-study comparability of positive margin and re-excision rates. Quality Assessment of Diagnostic Accuracy Studies-2 (QUADAS-2) and Risk of Bias in Nonrandomized Studies-of Interventions (ROBINS-I) would be the appropriate instruments for formal appraisal of the diagnostic accuracy and nonrandomized comparative studies, respectively; their application was outside the scope of this narrative review. The findings summarized here should accordingly be read as associations reported in observational settings rather than as demonstrated causal effects.

Training, credentialing, and implementation

Surgeon-performed POCUS is operator-dependent and requires structured, progressive training [21]. Evidence from procedural ultrasound curricula places the learning curve at 50 to 100 supervised examinations before reliable independent diagnostic performance is achieved, although this threshold varies with the operator’s prior imaging background and the complexity of the lesion mix [21]. The American Society of Breast Surgeons (ASBrS) has published practice guidelines defining minimum examination volumes, documentation standards, and competency assessment criteria [21]. Competency-based training programs that sequence didactic instruction, simulation-based scanning on phantoms, and mentored clinical practice followed by formative and summative evaluation represent the reference implementation model.

Credentialing pathways vary by geography. In the United States, the ASBrS offers a formal certification route requiring demonstrated training volume and documented case logs [21]. In Europe, credentialing structures differ by country and specialty society [16]. Quality assurance must be ongoing rather than a single gatekeeping exercise. Auditing diagnostic concordance with final pathology, reviewing biopsy guidance outcomes, and maintaining peer review of documentation standards are minimum requirements for a credible surgeon-performed POCUS program.

Barriers to adoption are substantial. Limited access to training programs, upfront capital investment in ultrasound equipment, medicolegal questions surrounding nonradiologist interpretation, and institutional inertia within conventional radiology-referral workflows collectively create friction [9,33]. In high-income settings, professional boundary disputes and credentialing gatekeeping can delay adoption even where clinical evidence supports an expanded surgical ultrasound scope. In LMICs, where dedicated radiology services are absent or intermittently available, portable surgeon-performed POCUS often functions as the primary diagnostic system rather than a supplement [22,33].

Limitations, challenges, and future directions

Operator dependency is the principal vulnerability of POCUS. Diagnostic accuracy tracks with training depth, clinical experience, and ongoing scanning volume [23,24]. Without standardized training pathways and robust quality assurance mechanisms, performance inconsistency creates patient safety risk through missed diagnoses and misinformed procedural decisions. The technique also has recognized scope boundaries: densely glandular breasts without supplemental imaging, mammographically occult lesions requiring stereotactic guidance, and high-risk patients requiring comprehensive multifocality mapping all expose limitations that surgeon-performed POCUS cannot resolve independently [30]. This review is itself subject to limitations. It was conducted as a narrative synthesis without a registered protocol, prespecified eligibility criteria, duplicate screening, or formal quality appraisal, and selection of the cited literature therefore reflects author judgment. Grey literature and conference abstracts were not searched systematically, and the possibility of publication bias favoring positive IOUS outcomes cannot be excluded.

Medicolegal and credentialing uncertainty remains a challenge in many jurisdictions, with variable definitions of scope of practice for surgical breast ultrasound and limited precedent regarding adverse outcomes in nonradiologist-performed studies [21]. Standardization of reporting formats, image archiving, and documentation practices is essential for quality and medicolegal accountability. The current evidence base is derived predominantly from single-institution retrospective studies and observational cohorts; prospective multicenter randomized trials comparing surgeon-performed with radiologist-performed breast ultrasound programs are needed.

AI may alter the operator-dependency equation. Deep learning-based computer-aided detection and diagnosis systems trained on breast ultrasound datasets have reported lesion characterization accuracy approaching expert radiologist performance in retrospective evaluations, generally without external validation or prospective testing at the point of care [19,20]. Embedded into point-of-care platforms, these tools could provide real-time BI-RADS scoring, automatic structured report generation, and decision-support flagging, raising the minimum acceptable diagnostic performance across operator experience levels [19,20]. Radiogenomic linkage connecting AI-extracted ultrasound imaging phenotypes to molecular tumor profiles raises the prospect of noninvasive tumor characterization and personalized treatment stratification without biopsy [20].

Hardware development follows a similar trajectory. Wireless, smartphone-compatible, palm-sized transducers are expanding POCUS accessibility in global health and low-resource contexts [9,22]. AI-enhanced models have demonstrated feasibility in predicting mammographic breast density from ultrasound images alone, a capability that could extend the diagnostic reach of point-of-care sonography [42]. Research priorities include prospective multicenter outcome comparisons, curricula validation studies, and formal health-economic analyses across diverse healthcare systems.

## Conclusions

Surgeon-performed POCUS is a purposeful clinical tool that bridges the separation between diagnostic imaging and operative decision-making rather than a substitute for unavailable radiology services. Real-time sonographic characterization at the point of care may support diagnostic assessment of complex breast lesions. The available evidence, drawn largely from single-institution observational studies, associates IOUS guidance with lower positive margin rates, fewer re-excision procedures, and favorable cosmetic outcomes in BCS; prospective multicenter comparative trials are required to confirm these findings. Integration into one-stop clinics compresses diagnostic timelines, improves the patient experience, and streamlines care coordination. Realizing the full potential of this approach requires investment in competency-based training, standardized credentialing, and institutional commitment to multidisciplinary integration. As AI-augmented platforms mature, portable hardware proliferates, and evidence-based protocols are established, surgeon-performed breast POCUS may assume a larger role in patient-centered breast care, contingent on evidence from prospective comparative studies.
